# Numerical simulation of realistic top coal caving intervals under different top coal thicknesses in longwall top coal caving working face

**DOI:** 10.1038/s41598-021-92624-y

**Published:** 2021-06-24

**Authors:** Chuang Liu, Huamin Li

**Affiliations:** 1 School of Resource and Safety Engineering, Henan University of Engineering, Zhengzhou, 451191 Henan China; 2 School of Energy Science and Engineering, Henan Polytechnic University, Jiaozuo, 454003 Henan China

**Keywords:** Geology, Fossil fuels

## Abstract

In the process of longwall top coal caving, the selection of the top coal caving interval along the advancing direction of the working face has an important effect on the top coal recovery. To explore a realistic top coal caving interval of the longwall top coal caving working face, longwall top coal caving panel 8202 in the Tongxin Coal Mine is used as an example, and 30 numerical simulation models are established by using Continuum-based Distinct Element Method simulation software to study the top coal recovery with 4.0 m, 8.0 m, 12.0 m, 16.0 m, 20.0 m and 24.0 m top coal thicknesses and 0.8 m, 1.0 m, 1.2 m, 1.6 m and 2.4 m top coal caving intervals. The results show that with an increase in the top coal caving interval, the single top coal caving amount increases. The top coal recovery is the highest with a 0.8 m top coal caving interval when the thickness of the top coal is 4.0 m, and it is the highest with a 1.2 m top coal caving interval when the coal seam thickness is greater than 4.0 m. These results provide a reference for the selection of a realistic top coal caving interval in thick coal seam caving mining.

## Introduction

Top coal caving mining technology started in the 1980s and continues to this day^1,2^. Longwall top coal caving (LTCC) mining consists of two processes: shearer cutting in front of a hydraulic support and top coal caving behind a hydraulic support. The parameters of the top coal caving interval (TCCI) in the process of top coal caving behind a hydraulic support have an important impact on the top coal recovery^3,4^. The top coal recovery is an important index to evaluate the success of top coal caving mining. Therefore, improving the top coal recovery is an important technical problem faced by LTCC mining^5–7^.

Wang et al. derived an equation of a possible caving ellipsoid and an actual caving ellipsoid on the basis of caving ellipsoid theory and analyzed the optimization calculation method of caving parameters along the dip direction of the working face^8–10^. Sun et al., using a similar simulation, constructed 15 coal drawing models according to different TCCIs, coal drawing sequences, coal drawing methods and coal seam dip angles; they compared and analyzed the influence of different coal drawing conditions on the top coal recovery and the movement of top coal and gangue, and they concluded that the top coal recovery of the working face was the highest when a single round sequential coal drawing method from bottom to top was adopted in the working face dip direction^11,12^. Li et al., with the method of orthogonal experimental design, used the top coal crushing coefficient as the index and the hydraulic support height, hydraulic support length and TCCI of the hydraulic support as the influencing factors to carry out range and variance analyses on the simulation results of PFC 2D and obtain the sensitivity of each influencing factor on the top coal recovery^13^.

Yang et al., by using the method of numerical simulation, analyze the top coal recovery rate of the dynamic group caving method (DGCM) and single-opening sequence caving method (SSCM) under different mining and caving ratios. When the mining caving ratio is less than 1:1, the recovery ratio of DGCM is obviously higher than that of SSCM, indicating that DGCM can greatly improve the recovery ratio. While when the mining-caving ratio is larger than 1:1, the recovery ratio of two caving methods is basically equal; however, the advancing time is significantly shortened under DGCM^14^.

Wang et al., using the integrated FDM–DFN model enabled the influence factors of the failure process of top coal. The brittle rupture of the main roof resulted in a sudden release of elastic strain energy, which transformed into kinetic energy of the caved blocks and resulted in the dynamic load in the LTCC face. The dynamic load facilitated the development of mining induced fractures in the top coal^15^.

Li et al., investigate the reasonable numerical damping for modeling top-coal caving by comparing with the experimental results. The result shows that the most reasonable numerical damping value is 0.07 for the numerical modeling of interval top-coal caving in extra-thick coal seams^16^.

Song et al., established a progressive-sectional failure model (PSFM) based on Particle Flow Code to simulate top coal drawing process in longwall top coal caving (LTCC) mining. The simulation results show that the top coal loss in PSFM is larger than loose granular model (LGM), and the top coal boundary in PSFM shows a “step” subsidence and is different from that in LGM^17^.

Based on Continuum-based Distinct Element Method (CDEM) numerical simulation software, this study uses the 8202 LTCC working face of the Tongxin Coal Mine. According to the top coal caving law along the strike direction of the LTCC working face, this paper establishes a numerical simulation model along the strike direction of the working face, systematically simulates the top coal recovery of the LTCC working face under different top coal thickness and TCCIs, and summarizes the best TCCI under different top coal thickness for the maximum top coal recovery according to the results of the numerical simulation.

## Numerical simulation model

### Establishment of the numerical simulation model

In this study, CDEM software is used to simulate the top coal caving during the mining process of panel 8202. The CDEM software was developed by the Institute of Mechanics, Chinese Academy of Sciences, and its algorithm couples the finite element method (FEM) and the discrete element method (DEM)^18^.

**Figure 1:**
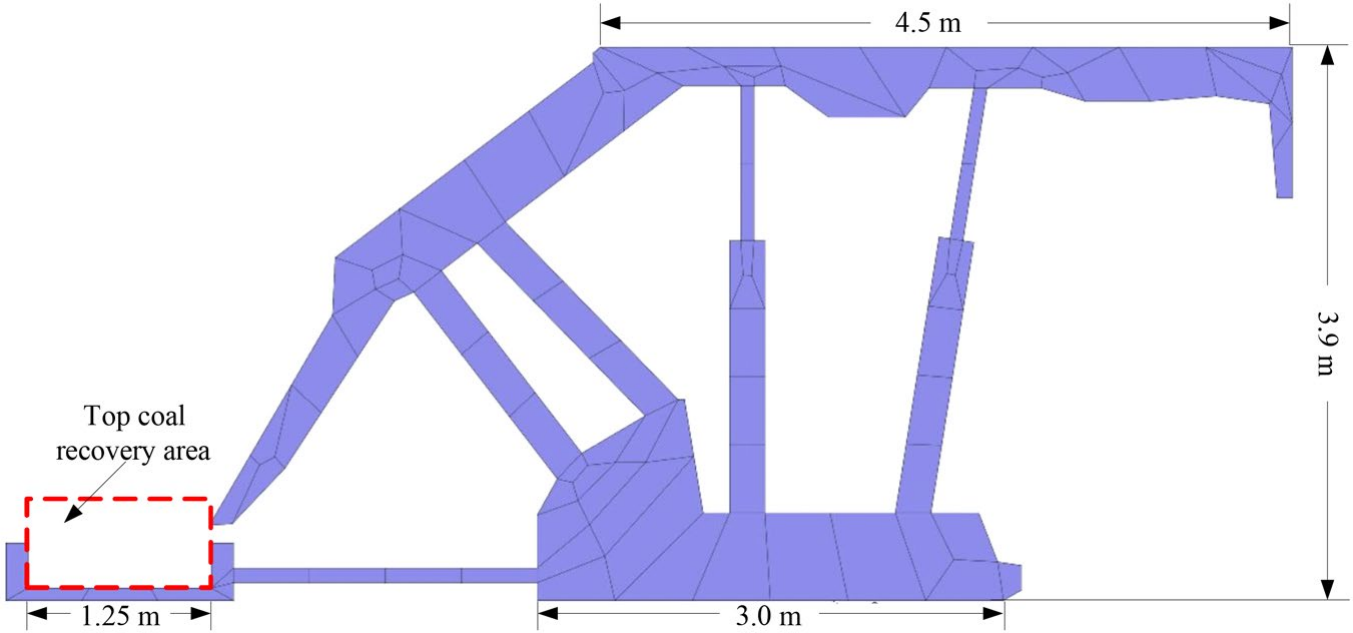
Model and size of shield used in numerical simulation.

To research the influence of the TCCI on the top coal recovery under different top coal thickness conditions, according to the specific size parameters of the ZF15000/27.5/42 low-level caving hydraulic support used in the 8202 LTCC working face of the Tongxin Coal Mine, simplified processing is first carried out to establish a two-dimensional numerical simulation model of the caving hydraulic support, as shown in Fig. 1.

**Figure 2:**
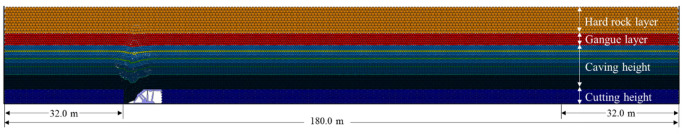
Numerical simulation model along the strike direction in LTCC working face.

The length of the numerical simulation model is 180.0 m, the reserved length of the left and right boundary is 32.0 m, and the left and right sides and the lower part of the model are fixed. In the model, the coal cutting height of the shearer is 3.9 m, and the top coal with different thicknesses is located above the shearer cutting coal seam. The established top coal thickness is 4.0 m, 8.0 m, 12.0 m, 16.0 m, 20.0 m and 24.0 m, the immediate roof (gangue) is 3.0 m thick, and the upper part of the immediate roof is 7.0 m thick and is composed of hard rock. The model is simulated from left to right, and the first 20.0 m of the simulation does not cave the top coal but only cuts the coal seam with a shearer cutting height. After 20.0 m, while the shearer cuts the coal seam, the top coal is recovered in the corresponding area of the rear scraper conveyor. During the simulation process, the gangue falling into the top coal area of the rear scraper conveyor is monitored as the standard to move the frame. That is, for the gangue moving frame, the established numerical simulation model is shown in Fig. 2.

### Model parameters

According to different forces on the top coal in different rock layers, the degree of particle fragmentation increases from top to bottom. The top coal particles of the established numerical simulation model decrease sequentially from top to bottom. The particle size setting of each rock layer in the model is shown in Table 1, and the mechanical parameters of the particles in the model are shown in Table 2.

**Table 1:** Particle diameter and height of the numerical model rock layer.

Rock layer	Diameter of particles (m)	Height (m)
Hard rock	0.4	7.0
Gangue	0.3	3.0
Upper top-coal	0.25	–
Middle upper top coal	0.2	–
Middle top coal	0.2	–
Middle lower top coal	0.15	–
Lower top coal	0.1	–
Cutting coal seam	0.2	3.9

**Table 2:** Particle mechanical parameters of coal and roof rock strata.

Rock layer	Unit weight (kN/m^3^)	Elasticity modulus (GPa)	Poisson’s ratio	Tensile strength (MPa)	Cohesion (MPa)	Friction angle (°)
3 ~ 5# coal seam	13.4	2.8	0.34	0.5	0	28.6
Carbon mudstone	15.6	4.4	0.29	0.9	15.9	33.7
Sandy mudstone	25.3	24.2	0.25	4.7	42.4	41.3

## Numerical simulation of the top coal caving interval

The selection of the TCCI in the LTCC working face has a great influence on the top coal recovery. Both too large and too small of a TCCI leads to a decrease in the top coal recovery. Based on the current LTCC working face, the shearer cutting depth is mainly 0.6 m and 0.8 m, while very few adopt 1.0 m, and the integer multiple of the shearer cutting depth is selected as the TCCI. A large TCCI is poorly applied in production practice because the top coal loss is substantial. Therefore, this paper focuses on the simulation of "one cutting and one caving" and "two cuttings and one caving" top coal caving intervals, that is, 0.8 m, 1.0 m, 1.2 m and 1.6 m top coal caving intervals.

For the large TCCI, only 2.4 m is simulated. This paper simulates and analyzes the top coal recovery of top coal with thicknesses of 4.0 m, 8.0 m, 12.0 m, 16.0 m, 20.0 m, and 24.0 m under 5 different TCCI conditions. Taking the top coal thicknesses of 4.0 m, 12.0 m, and 20.0 m as examples, the effect of the TCCI on the top coal recovery under different top coal thickness conditions is explained in detail.

### Simulation of the top coal recovery of a 4.0 m thickness under different top coal caving intervals

**Figure 3:**
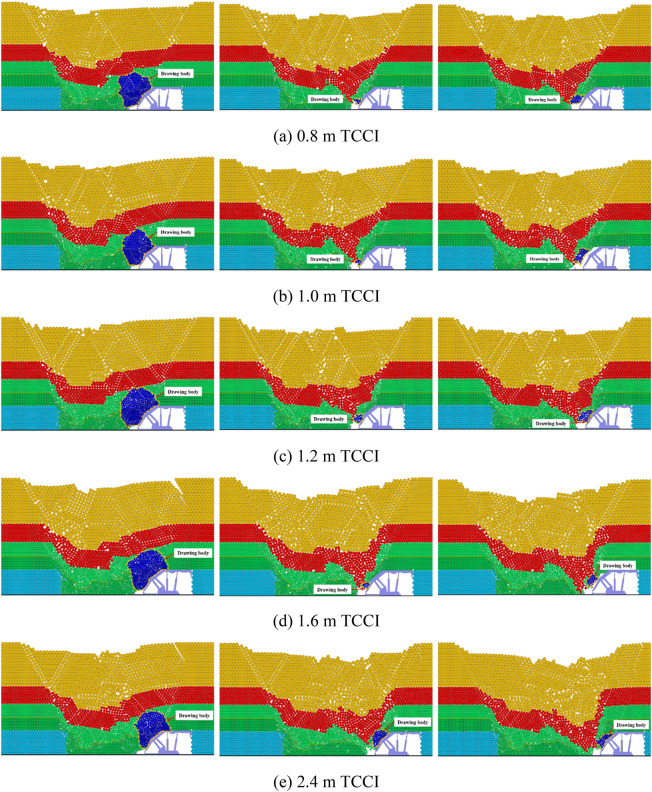
Inversion results of the first three top coal release body in different coal-drawing interval under 4.0 m top coal thickness.

Figure 3 shows the inversion results of the first three steps of the top coal caving bodies of the 4.0 m thick top coal under different TCCIs. Figure 3 shows that there is no obvious difference in the first three steps of the top coal recovery of 4.0 m thick top coal under different TCCIs. When there is no gangue boundary on the left side of the caving window, the first step of the top coal caving amount under different TCCI conditions is relatively large, and the caving body is cut into an oval-like shape.

In the subsequent second and third top coal caving steps, due to the large top coal caving amount in the first step, the gangue above the top coal moves to the vicinity of the top coal caving window. After the first top coal caving is completed, the gangue is close to the upper left of the top coal caving window. When the top coal caving window is opened and the top coal is cleaved again, it is easy to cause the gangue near the top coal caving window to mix in advance and end the current top coal caving process. Therefore, the recoveries in the second and third top coal caving steps are greatly reduced compared with the recovery in the first top coal caving step. Only a few top coal particles are recovered before the gangue reaches the recovery area of the rear scraper conveyor, and there is no obvious shape of the top coal drawing body.

**Figure 4:**
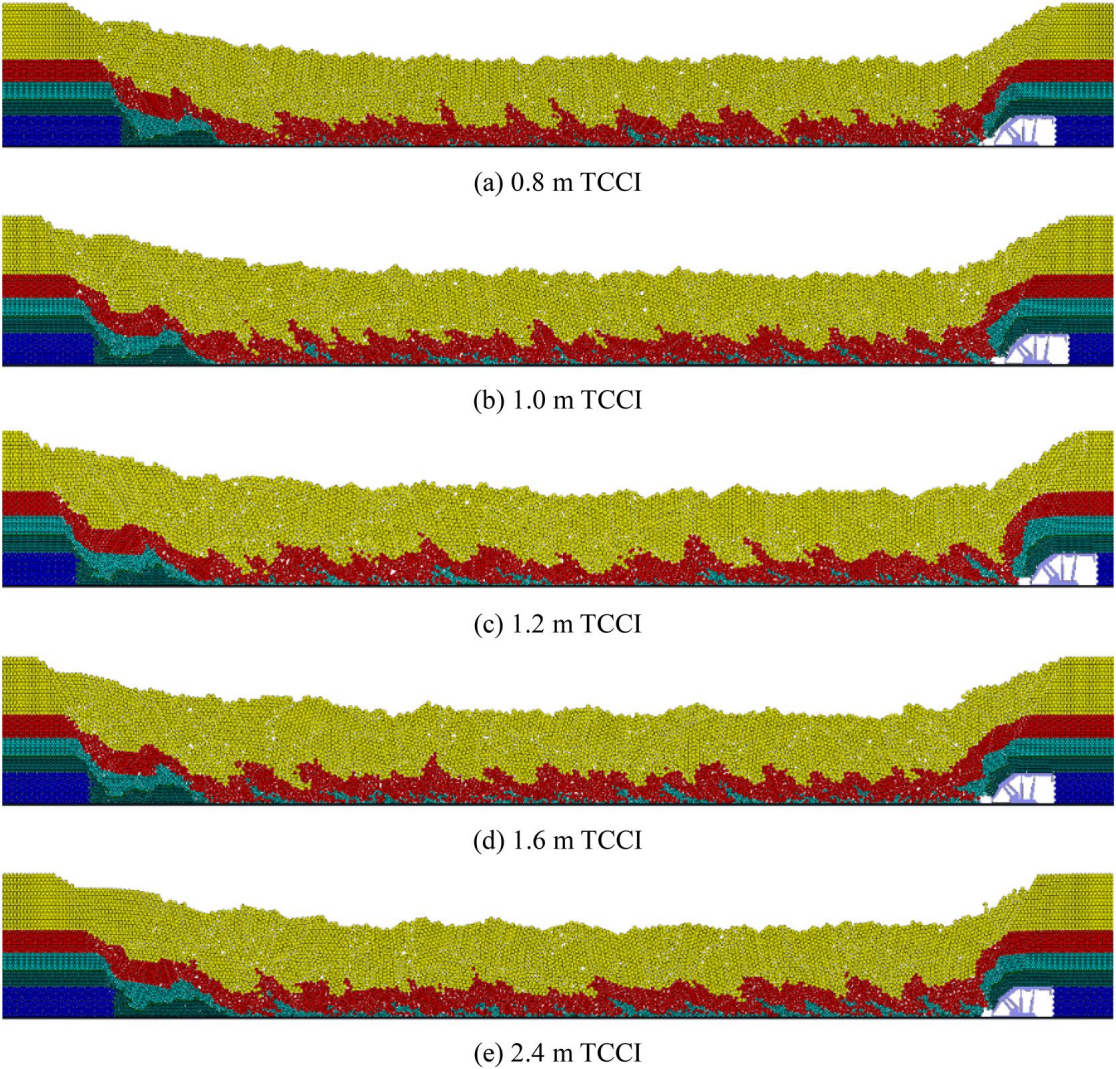
Loss of top coal in different coal-drawing interval under 4.0 m top coal thickness.

The top coal loss and coal-gangue interbeds of the 4.0 m thick top coal under different TCCI conditions are organized as shown in Fig. 4. Under different TCCI conditions, the top coal loss and coal gangue interbeds are different. Figure 4 clearly shows that less top coal is lost and the coal-gangue interbeds are thinner under the 0.8 m TCCI. The most top coal is lost under the 2.4 m TCCI. Under different TCCIs, the descending gangue interface is jagged with no fixed shape.

### Simulation of the top coal recovery of a 12.0 m thickness under different top coal caving intervals

**Figure 5:**
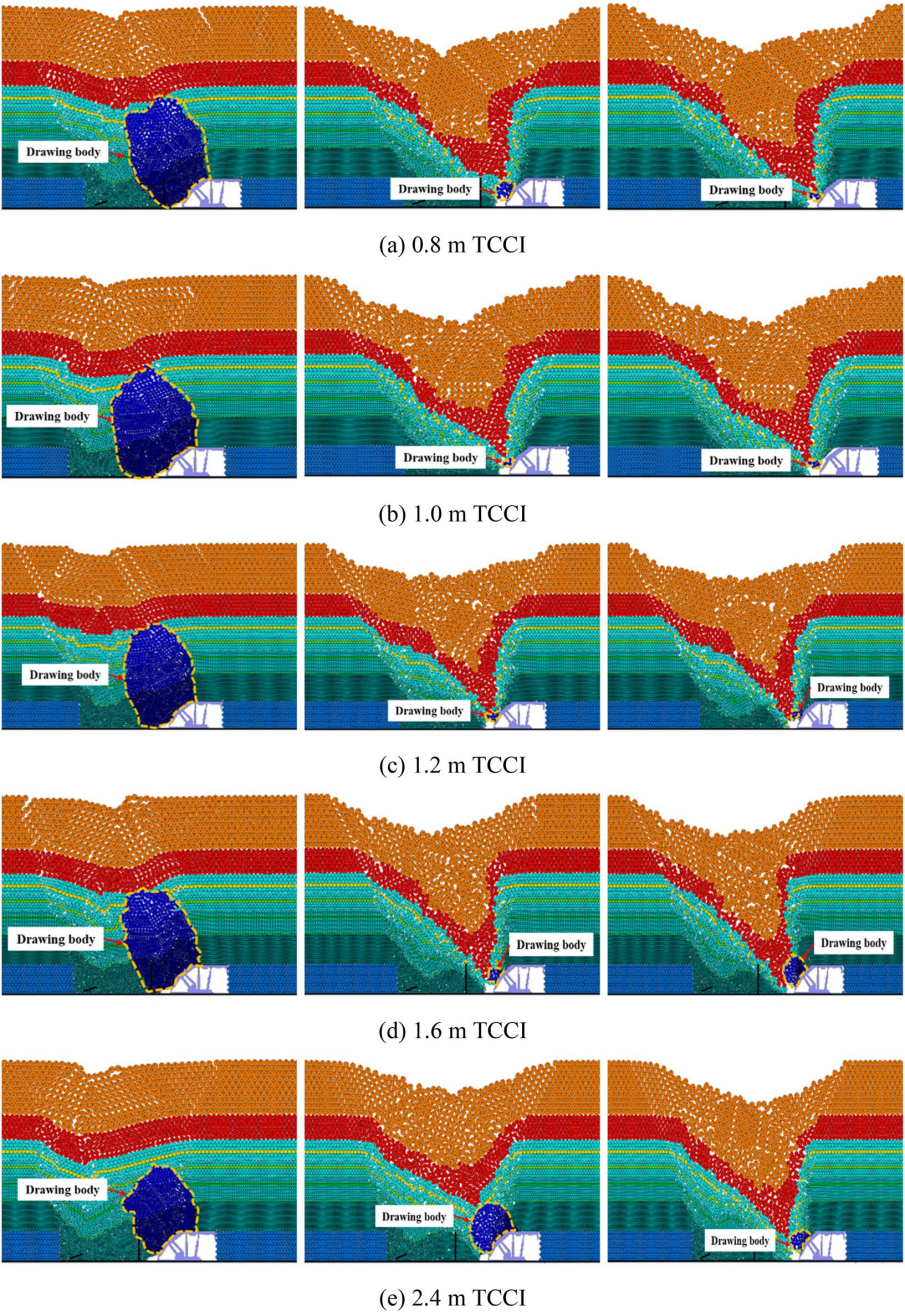
Inversion results of the first three top coal release body in different coal-drawing interval under 12.0 m top coal thickness.

The inversion results of the first three steps of top coal caving bodies with 12.0 m thick top coal intervals under different TCCI conditions are shown in Fig. 5. The top coal recoveries of the 12.0 m thick top coal and 4.0 m thick top coal in the first three steps are basically the same. The top coal recovered in the first step is large, and the shape of the discharge body is an ellipse. The top coal caving amounts recovered in the second and third steps are small, and there is no obvious discharge body shape. In the simulation of the 2.4 m TCCI, the phenomenon of the top coal arching above the caving window appears in the first top coal caving process, which blocks the caving window, resulting in a relatively small top coal recovery. The relative amount of top coal recovered during the second and third caving operations increases. Under different TCCIs, except that the top coal recovery of the 2.4 m TCCI is different from other groups due to the arch formation of the top coal, there is no obvious difference in the first three steps of the top coal recovery of other groups.

**Figure 6:**
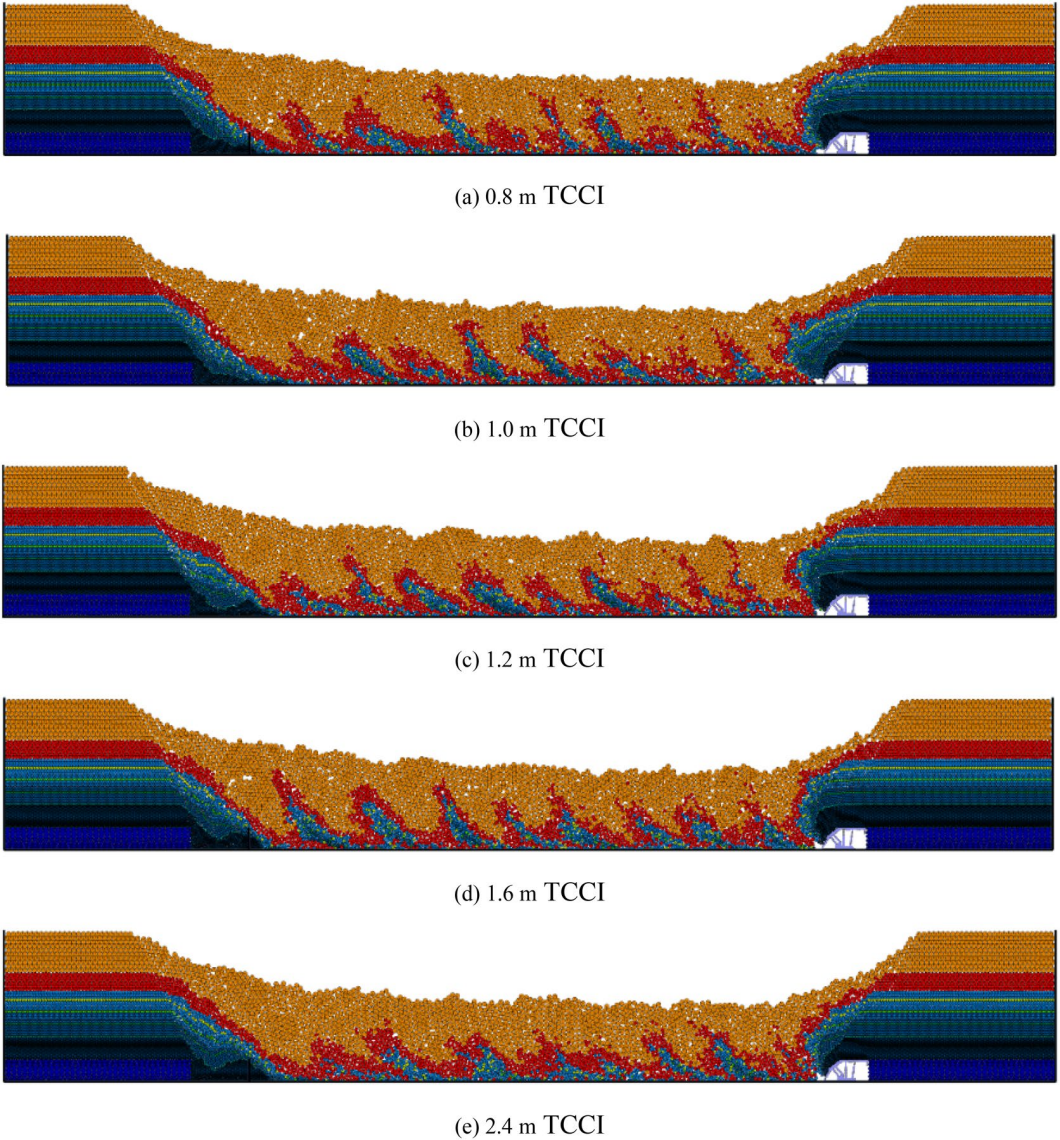
Loss of top coal in different coal-drawing interval under 12.0 m top coal thickness.

The top coal loss and coal-gangue interbeds of the 12.0 m thick top coal under different TCCIs are shown in Fig. 6. Compared with the coal-gangue interbeds of the 4.0 m thick top coal under different TCCIs, the coal-gangue interbed interface of the 12.0 m thick top coal is obvious, and the horizontal distance between two adjacent coal-gangue interbedding planes is 10–12 m. The top coal loss under the 0.8 m, 1.0 m and 1.2 m TCCIs is obviously less than that under the 1.6 m and 2.4 m TCCIs.

### Simulation of the top coal recovery of a 20.0 m thickness under different top coal caving intervals

**Figure 7:**
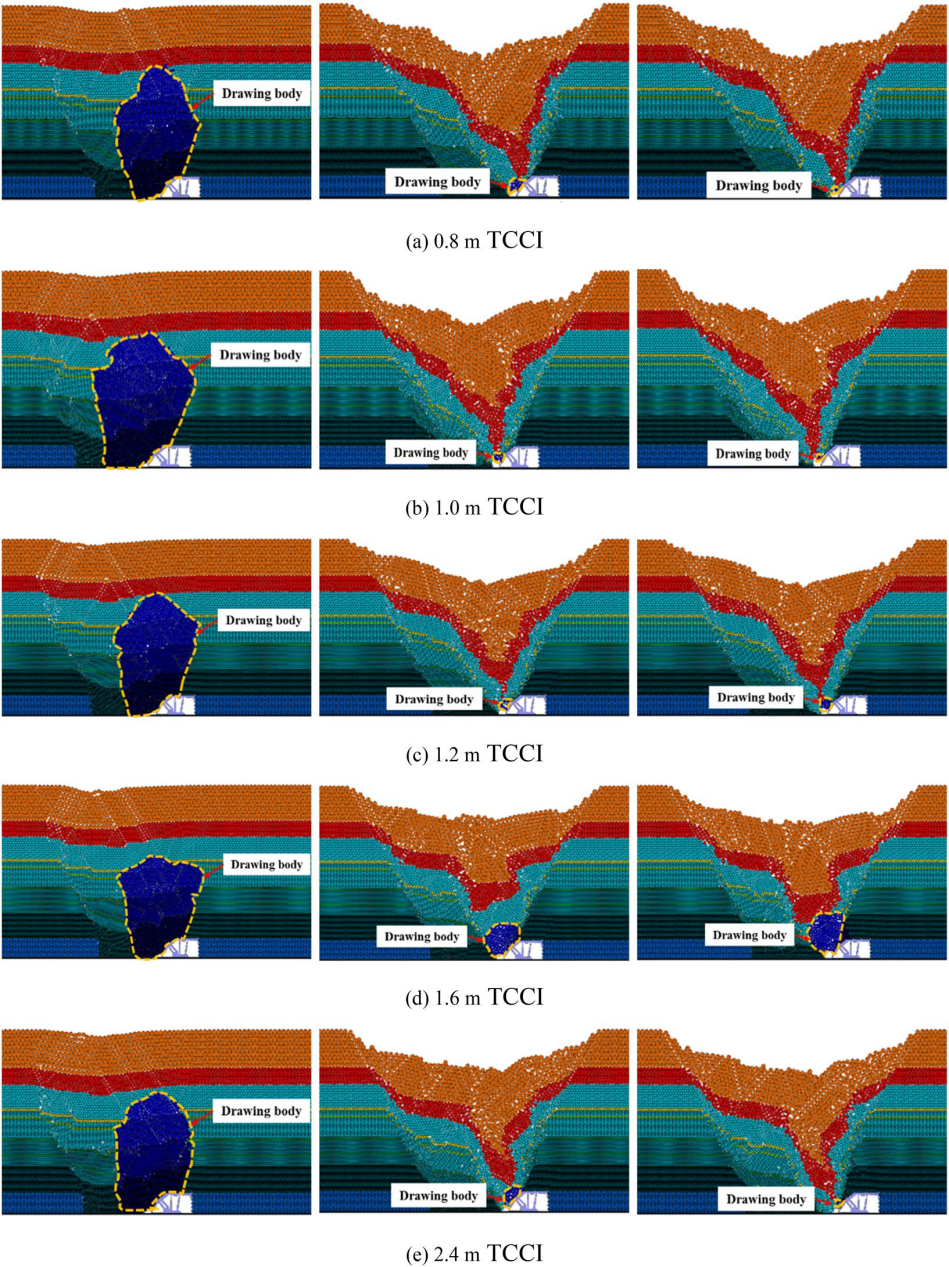
Inversion results of the first three top coal release body in different coal-drawing interval under 20.0 m top coal thickness.

Figure 7 shows the inversion results of the top coal caving bodies with a thickness of 20.0 m under different TCCIs. The thickness of the 20.0 m top coal is relatively large, and the top coal transportation trajectory is long. Compared with the top coal thicknesses of 4.0 m and 12.0 m, a coal arch above the caving window more easily forms under a thickness of 20.0 m, blocking the caving window and making the top coal unable to be recovered smoothly. Different degrees of top coal arching occur during the first coal caving step of thicknesses of 1.6 m and 2.4 m TCCIs, which reduces the top coal recovery. Under the 1.6 m and 2.4 m thick TCCI conditions, the recovery of the top coal in the second and third caving steps increases. The recovery and shape of the top coal drawing body under the conditions of the other TCCI groups are similar.

**Figure 8:**
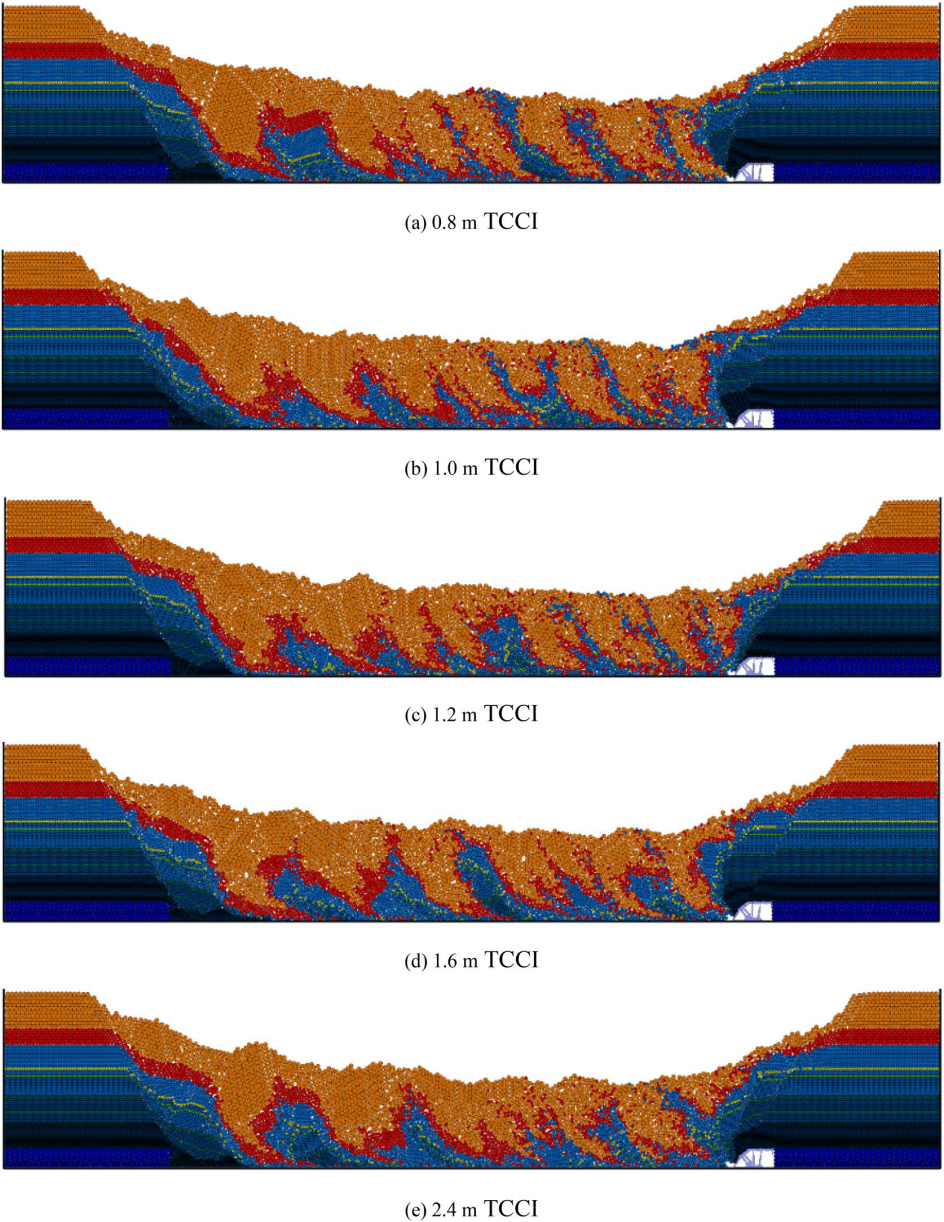
Loss of top coal in different coal-drawing interval under 20.0 m top coal thickness.

The top coal loss and coal-gangue interbeds of 20.0 m thick top coal under different TCCIs are shown in Fig. 8. Due to the large thickness of the top coal and the long interface of the coal-gangue interbeds, during the caving process, the gangue above the caving window easily reaches the caving window before it reaches the top coal above the caving window, leading to the end of top coal caving. Therefore, the absolute amount of top coal loss is relatively large in the process of thick top coal caving. The horizontal distance between two adjacent coal gangue interlayers is 16–20 m. Figure 8 also shows that the top coal loss under the 1.2 m thick TCCI is significantly less than that of the other four groups, and the top coal losses under the 1.6 m and 2.4 m thick TCCIs are the largest.

## Statistical calculation of the top coal recovery

**Figure 9:**
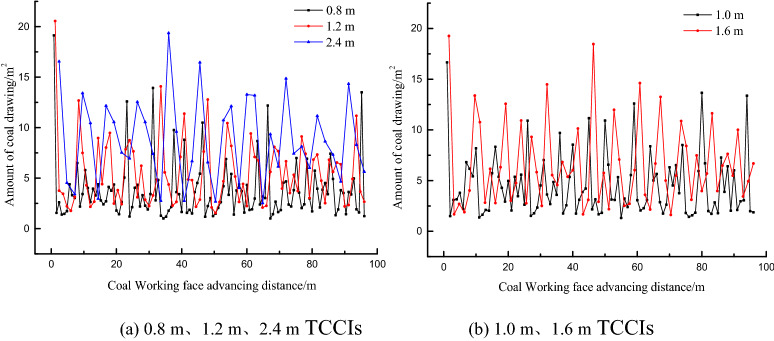
Statistics of top coal recovery amount of each step in different coal-drawing interval under 4.0 m top coal thickness.

**Figure 10:**
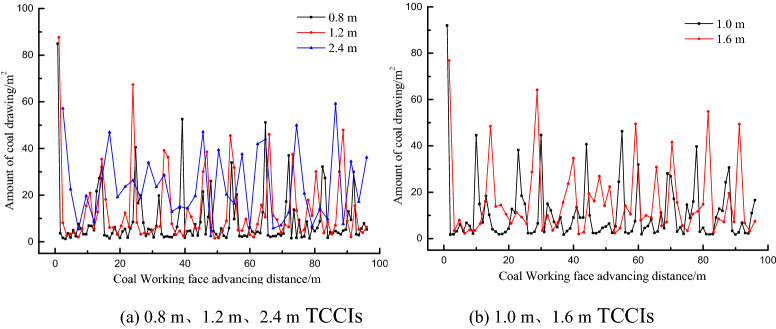
Statistics of top coal recovery amount of each step in different coal-drawing interval under 12.0 m top coal thickness.

**Figure 11:**
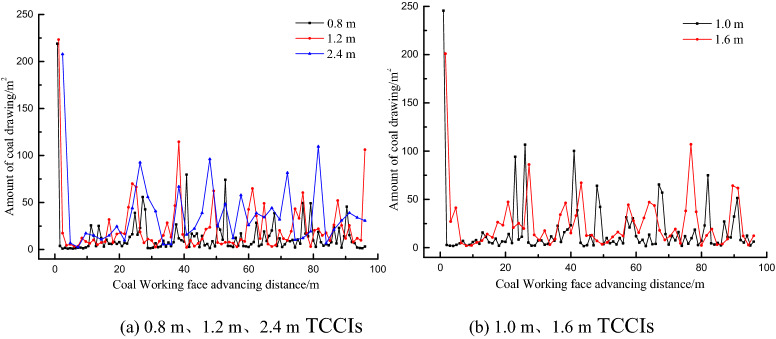
Statistics of top coal recovery amount of each step in different coal-drawing interval under 20.0 m top coal thickness.

To understand the recovery of the top coal in each top coal caving step under different TCCIs, the top coal thicknesses of 4.0 m, 12.0 m and 20.0 m were calculated. The recovery amount of top coal in a caving step is shown in Figs. 9, 10 and 11. The top coal recovery in a single caving step under various top coal thicknesses generally increases with increasing TCCIs. For the same top coal thickness, the dispersion degree of the top coal recovery is different in different TCCIs. The mean square deviation of the top coal recovery of the 4.0 m thick top coal is 2.90, 3.11, 3.38, 4.19 and 4.27 under the thicknesses of 0.8 m, 1.0 m, 1.2 m, 1.6 m and 2.4 m TCCI, respectively. The mean square deviation of the top coal recovery of the 12.0 m thick top coal is 12.23, 13.64, 15.64, 16.37, and 15.10; the mean square deviation of the top coal recovery of the 20.0 m thick top coal is 23.40, 31.48, 31.41, 31.22, and 37.55. The mean square deviation of the top coal drawing amount in each caving step increases with the increasing TCCI and top coal thickness; that is, the larger the TCCI and top coal thickness are, the more unbalanced the top coal recovery of each top coal caving window is.

**Figure 12:**
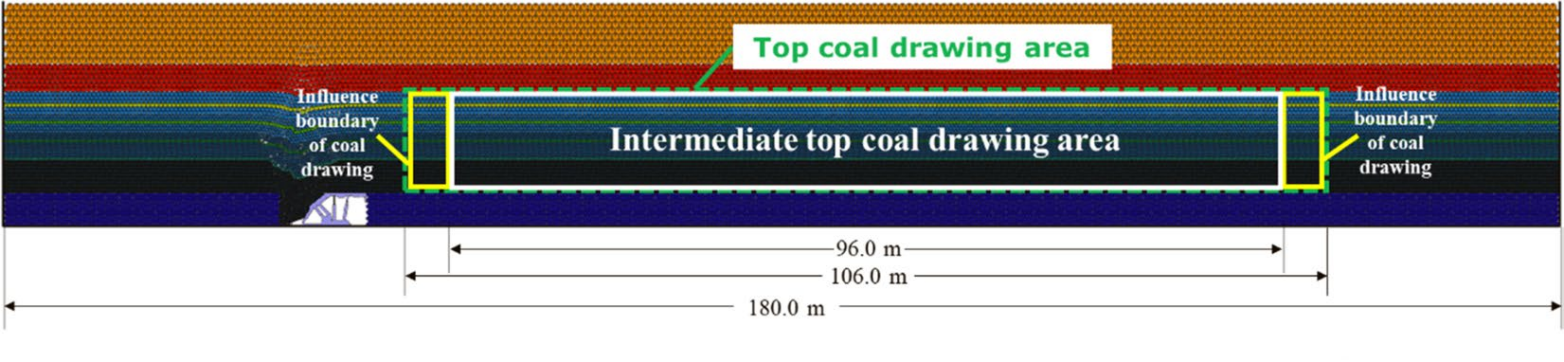
Schematic diagram of the total top coal caving area in the statistics of top coal caving along the strike direction of working face.

**Figure 13:**
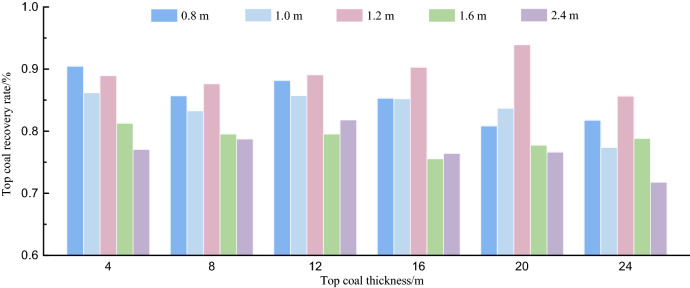
Statistic of top coal recovery rate under different coal-drawing interval and top coal thickness.

To compare the top coal recovery of different top coal thicknesses under different TCCIs, it is necessary to count and calculate the top coal recovery in the process of top coal caving. The total area of the top coal is calculated by the number of top coal particles in the caving area and the boundary area of 5.0 m affected by the caving on the left and right sides, as shown in Fig. 12. The top coal recovery is the ratio of the total area of the top coal particles falling into the rear scraper conveyor to the total area of the top coal. The top coal recoveries of 4.0 m, 8.0 m, 12.0 m, 16.0 m, 20.0 m and 24.0 m top coal thicknesses under different TCCIs are calculated. The statistical results are shown in Fig. 13.

In Fig. 13, the optimal TCCI for the top coal thickness of 4.0 m is 0.8 m, and the optimal TCCI for the remaining top coal thickness is 1.2 m. Among the TCCIs, the top coal recovery of the 4.0 m thick top coal under the 1.2 m TCCI is only 1.53% lower than that of the 0.8 m thick top coal. Under the 1.2 m thick TCCI, with the increase in the top coal thickness, the recovery of the top coal increases more obviously compared with other TCCIs. At present, the cutting depth of the shearer in the LTCC working face is typically 0.8 m, and the TCCI can be 0.8 m, 1.6 m and 2.4 m. According to the simulation results, the best TCCI is 0.8 m. If the TCCI can be increased to 1.2 m, the top coal recovery can be further improved.

## Conclusion

According to the geological conditions of the 8202 fully mechanized caving face in the Tongxin Coal Mine, a numerical simulation model along the advancing direction of the working face is established, and the different TCCIs of the LTCC working face are simulated and compared. The main conclusions are as follows.

1. The amount of top coal recovered in a single top coal caving step under different top coal thicknesses generally increases with increasing TCCIs.
2. After top coal caving under different top coal thicknesses, the coal-rock interface has an irregular jagged shape.
3. By simulating the top coal caving process with different top coal thicknesses of 0.8 m, 1.0 m, 1.2 m, 1.6 m and 2.4 m, it is concluded that the mean square deviation of the top coal drawing amount in each unit of distance moved generally increases with the increase in the TCCI and top coal thickness; this indicates that the smaller the TCCI and the thickness of the top coal, the smaller the difference in the amount of top coal recovered in each caving window, and the larger the TCCI and the top coal thickness is, the greater the difference in the caving amount in each caving window is.
4. Under different top coal thickness conditions, when the thickness of the top coal is 4.0 m and the TCCI is 0.8 m, the top coal recovery is the highest; when the coal seam thickness is greater than 4.0 m and the TCCI is 1.2 m, the top coal recovery is the highest.
5. With the continuous development of mechanized equipment in working face, especially the support capacity of hydraulic support, the width of scraper conveyor and the mining height of LTCC working face increase, the top coal caving ratio may break through the limit of 1:3, and the optimization of top coal caving interval will more consider the cooperation with mechanical equipment.
